# The solvability of quantum *k*-pair network in a measurement-based way

**DOI:** 10.1038/s41598-017-16272-x

**Published:** 2017-12-01

**Authors:** Jing Li, Gang Xu, Xiu-Bo Chen, Zhiguo Qu, Xin-Xin Niu, Yi-Xian Yang

**Affiliations:** 1 Information Security Center, State Key Laboratory of Networking and Switching Technology, Beijing University of Posts and Telecommunications, Beijing, 100876 China; 2 School of Software Engineering, Beijing University of Posts and Telecommunications, Beijing, 100876 China; 3 Jiangsu Engineering Center of Network Monitoring, Nanjing University of Information Science and Technology, Nanjing, 210044 China; 4 GuiZhou University, Guizhou Provincial Key Laboratory of Public Big Data, Guizhou, Guiyang 550025 China

## Abstract

Network coding is an effective means to enhance the communication efficiency. The characterization of network solvability is one of the most important topic in this field. However, for general network, the solvability conditions are still a challenge. In this paper, we consider the solvability of general quantum *k*-pair network in measurement-based framework. For the first time, a detailed account of measurement-based quantum network coding(MB-QNC) is specified systematically. Differing from existing coding schemes, single qubit measurements on a pre-shared graph state are the only allowed coding operations. Since no control operations are concluded, it makes MB-QNC schemes more feasible. Further, the sufficient conditions formulating by eigenvalue equations and stabilizer matrix are presented, which build an unambiguous relation among the solvability and the general network. And this result can also analyze the feasibility of sharing *k* EPR pairs task in large-scale networks. Finally, in the presence of noise, we analyze the advantage of MB-QNC in contrast to gate-based way. By an instance network ${G}_{k}$, we show that MB-QNC allows higher error thresholds. Specially, for *X* error, the error threshold is about 30% higher than 10% in gate-based way. In addition, the specific expressions of fidelity subject to some constraint conditions are given.

## Introduction

Quantum communication has become one of the most potential applications in quantum information since Bennet and Brassard have proposed a famous quantum key distribution (QKD) scheme in 1984^1^. Recently, point-to-point quantum communication is extending to quantum network^2,3^ where nodes with quantum storage and processing power are connected by quantum channels to meet the demands with high capacity(the amount of messages transmitted per network use), long distance and networking. Quantum components, like QKD whose original idea is to create a string of random private key between two distant parties securely, have been even realized in network^4,5^. Quantum secure direct communication(QSDC)^6^ in which secure message can be sent through quantum channel directly and need not to pre-share a private key are also generalized to accomplish the task in multi-parties^7,8^. This trend is also pushing other branches forward, such as quantum secret sharing(QSS)^9,10^, quantum teleportation(QT)^11–13^, quantum dense coding^14,15^ and other sub-branches based on them^16–21^. In this context, because of the complexity of network structure, a difficult problem is how to efficiently transmit quantum information against channel or local nodes errors such as the bottleneck problem caused by links collision. Or we can conclude this problem as the network “solvability” for a given communication task usually with capacity constraints.

Network coding^22,23^ refers to perform coding operations (consist of the combination operations such as exclusive-or operation and replicate of the received classical bits) on intermediate nodes to achieve higher capacity, load balancing and lower computation complexity. As an underlying tool, it is proved to be an effective means for communication efficiency in all kinds of practical applications^24–27^. Note that, here, we call network coding especially for the set up transmitting classical bits as classical network coding(CNC). Zhou designed an efficiency and accuracy near-duplicate elimination approach for visual sensor networks^24^. A fast motion estimation method is proposed to reduce the encoding complexity to 20% time saving^25^. For reducing the computational complexity, a content similarity based fast reference frame selection algorithm is also proposed^26^. And some optimal cluster-based mechanisms are proved to be efficient for load balancing with multiple mobile sinks for these problems^27^.

**Figure 1:**
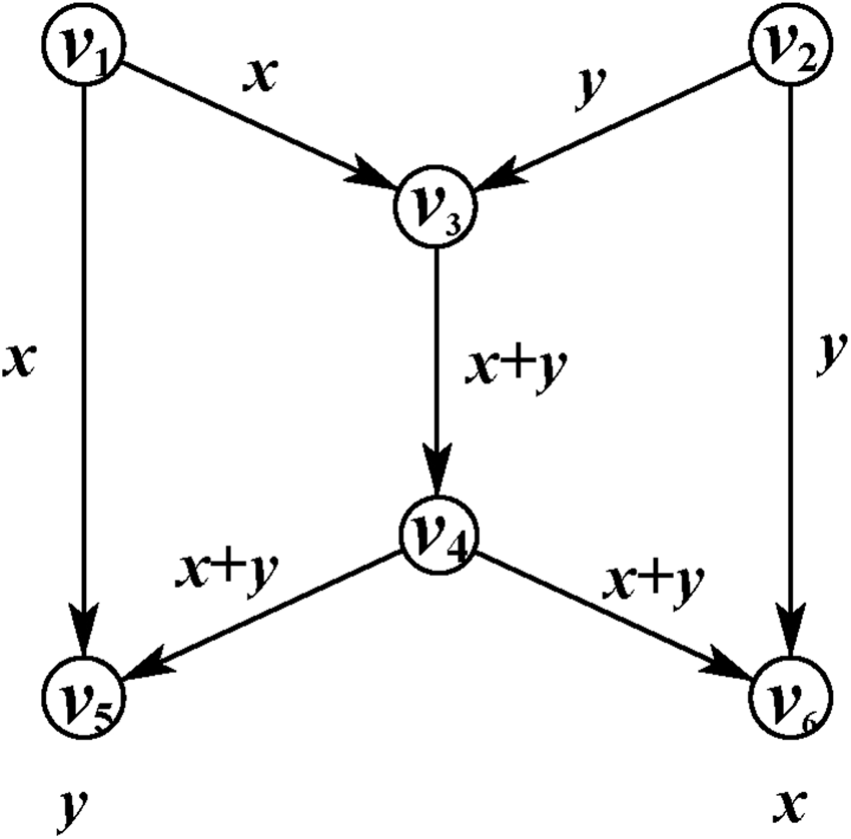
Butterfly network.

For the theoretical researches, many good results have been made. A simple and outstanding example is butterfly network (a typical 2-pair network) as shown in Fig. 1 in which two bits can be transmitted simultaneously from ${v}_{1}$ to ${v}_{6}$ and ${v}_{2}$ to ${v}_{5}$ via exclusive-or operation on ${C}_{1}$, ${T}_{1}$ and ${T}_{2}$. This capacity is generally not accomplished because of the capacity constraints in common channels $({C}_{1},{C}_{2})$. A network is *solvable* if there exists a network coding scheme (a choice of coding operations, also called *solution*)such that a given communication task is achievable. The solvability consider the question whether a given network is solvable for specific communication task with network coding. And two kinds network coding problems are mainly concerned: multicast problem^22^ and multi-unicast problem (*k*-pair problem)^23^. In its first stage, multicast problem where all messages of the only source are needed by all sinks attracted wide attentions and effective solutions have been obtained by the min-cut/max-flow condition^22^ and linear coding operations^28^. The *k*-pair problem is another important branch in network coding where the message of each source is only needed by its corresponding sink. The solvability is more complicated than multicast network problem. Researchers attempt to either design constructive coding schemes or present the necessary and/or sufficient conditions^29,30^ for some specific networks. However, for more general *k*-pair network, it is still an open challenge.

In this paper, our work is closest in spirit to these papers. Specifically, we also examine the solvability of general *k*-pair network. However, they are some essential differences in the problem setting which give rise to a new field–Quantum network coding(QNC). A typical difference is cloning. Classical bits can be accurately copied arbitrarily but it is not possible exactly for quantum bits because of quantum no cloning theorem^31^. In addition, different from classical butterfly network, it has proved that two qubits perfect transmission is not possible without any assistant resource for quantum butterfly network^32,33^. Consequently, QNC is not a simple repetition of CNC, but an important subject need to further study.

The study on the solvability of quantum *k*-pair network got going in 2006^32^. Hayashi *et al*. firstly shown the butterfly network is solvable with fidelity strictly greater than 0.5. To analyze the solvability of specific network and to design constructive schemes with lower communication cost are still two major issues^34^. It is well known that the network solvability is closely related to its topology structure, and for general *k*-pair network, its determinant is still a challenge. Related to these studies, entanglement^33,35–39^ is introduced to explore this problem. Kobayashi *et al*.^35,36^ presented the sufficient condition for a class of *k*-pair network in gate-based framework. We call network coding as Gate-based QNC (GB-QNC) where the coding operations consisting of projective measurements and coherent control gates are performed node-by-node by simulating the coding scheme solving the classical problem. It is also proved to be effective in entanglement distribution^40,41^. In fact, The essence of its encoding process is to create entanglement. Therefore, there is no obvious boundaries between the entanglement creation and its use as a resource making the relationship between the solvability and network structure misty. It is worth mentioning that, Beaudrap^42^ demonstrated that GB-QNC^35,36^ is in fact a special case of measurement-based procedure^43^. In MB-QNC, coding operation consists of local (projective) measurements on a highly entangled resource graph state which are responsible for the transmission of quantum bits. In this sense, it also offers a potential experimental advantage since no coherent operations are concluded. MB-QNC which can systematize the coding operations is a desirable way to design efficient communication schemes. The solvability of some special networks in Measurement-based have been examined^39^. However, no solvability conditions are presented yet for general *k*-pair network.

One contribution of this paper is to present sufficient conditions for the solvability with MB-QNC for the first time. To start with the correlation between network structures and graph states, the general process of MB-QNC is systematically specified. In contrast to the standard GB-QNC, no entanglement is created during the computation, we thus have a clear distinction between the entanglement creation and its use as a resource. Secondly, by reducing *k*-pair task to a specific clifford group operation, we build an unambiguous functional relationship between the solvability and the network structure with 2*k* eigenvalue equations. Further, for the question how to construct coding scheme? We present another sufficient condition given by the stabilizer matrix. This conclusion can also analyze the feasibility in the communication task to sharing *k* EPR pairs over arbitrary networks.

The works above are mainly focused on the noiseless resource states and the perfect quantum operations. In 2016, Satoh^44^ find that GB-QNC is more sensitive to noises especially on control operations. Specially, for pauli *X* and *Z* error, they prove that final fidelity would drop below 0.5 if the initial resource error is 10%. Motivated by these works, the performance of QNC in presence of noise is another concern in this paper. A second contribution of this paper is that we show the performance advantage of MB-QNC compared with GB-QNC. We will use a heuristic local Pauli-diagonal-noise channels to describe both errors. Since fidelity is usually difficult to calculate for complex network, we will work in stabilizer basis representation of mixed graph states by which the evolution of graph states can be easily tracked and just keeps them in diagonal form. Further, we present a set of constraint conditions to obtain more simpler expressions for fidelity in noisy resource error, noisy measurement error and both of them cases respectively. The property that multiple noises on the same qubit are equal to its the linear superposition makes us summarize all noises effects on resource state noise only. Finally, we apply above results to an instance butterfly network, showing that MB-QNC allows higher error threshold compared with the GB-QNC.

## Results

### Network Setting

The basic set up of a k-pair network is as follows. Specially, a *general k-pair network* here refers to a finite, directed and acyclic graph ${G}_{RK}=(V,E)$, where $V$ is the set of nodes or vertices, and $E$ is the set of edges, whose elements are pairs of nodes that are adjacent. The set of its *k* source nodes is $In({G}_{RK})=\{{v}_{1},\cdots ,{v}_{k}\}$, *k* sink nodes is $O({G}_{RK})=\{{v}_{k+n+1},\cdots ,{v}_{2k+n}\}$ and arbitrary $n$ intermediate nodes is $M({G}_{RK})=\{{v}_{k+1},\cdots ,{v}_{k+n}\}$. The quantum operations on each node are defined by any trace-preserving completely positive(CP-TP) map. The goal in any given *k*-pair network is to communicate *k* independent messages simultaneously from each source to its corresponding sink. Clearly, this task depends on the particular properties of the network and it might or might not be possible to achieve for the given *G*, *S*, and *T*. We shall be concerned with cases where the actual network topology given by *G* does not allow disjoint paths between the qubits in *S* and the qubits in *T*, but we nevertheless want to achieve perfect state transfer. Let ${\mathcal H} ={C}^{2}$ be a Hilbert space where *C* is complex field, we say ${G}_{RK}$ is quantum *solvable with fidelity* $F$ if there is choice of quantum operations(QNC scheme) over all nodes allowing us to send a quantum state $|{\psi }_{1}\rangle \otimes \cdots \otimes |{\psi }_{k}\rangle \in { {\mathcal H} }^{\otimes k}$ supported on the source nodes to the sink nodes with fidelity at least *F*. In particular, when *F* = 1, it is simply called *(perfect) solvable*. Here we consider MB-QNC local projective measurements on a highly entangled resource graph state associated to the network are responsible for the transmission of quantum bits.

**Figure 2:**
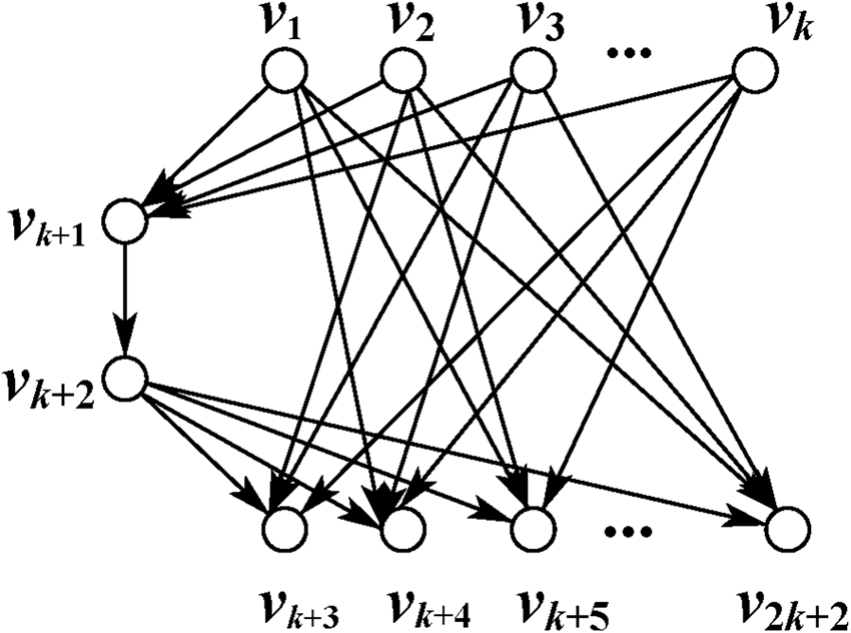
*G*_*k*_ network.

Among all research hotspots in *k*-pair network, Butterfly network and *G*_*k*_ network keep appearing in a high frequency, as denote in Figs 1 and 2.

### Graph state and its properties

Now, we review the concept of graph state, describe some of its properties, and fix the notation. It is sufficient to describe graph states with undirected finite graphs since the interaction of pairs of qubits are not necessarily bound up with the direction of edges. A undirected finite graph $G=\{V(G),E(G)\}$ is an ordered pair of sets: the set $V(G)$ of nodes or vertices, and the set $E(G)$ of edges, whose elements are pairs of nodes that are adjacent. In the following, we will mainly consider simple graph which contains neither loops (edges connecting nodes with itself) nor multiple edges between the same vertices. The set of nodes adjacent with ${v}_{i}$ is denoted by $N({v}_{i})=\{{v}_{j}:({v}_{i},{v}_{j})\in E(G)\}$, simply by $N({v}_{i})$, the set of other nodes $O({v}_{i})=V(G)-\{{v}_{i}\}\cup N({v}_{i})$ is simply denoted by $O({v}_{i})$ and the degree $|N({v}_{i})|$ is the number of nodes in $N({v}_{i})$. The adjacency relation gives rise to an *adjacency matrix* $\Lambda =({\lambda }_{ij})$, where ${\lambda }_{ij}=1$, if ${v}_{j}\in N({v}_{i})$, otherwise ${\lambda }_{ij}=0$. The adjacency relation of node ${v}_{i}$ can be represent by *adjacency vector* ${{\boldsymbol{v}}}_{i}$.

A graph state^45^ $|G\rangle$ is a quantum state associated with a graph *G*, where vertices correspond to qubits and edges to interactions. A common description of graph states by stabilizer formalism is described as follows. For a graph *G*, there are $n=|V(G)|$ stabilizer operators (one for each vertex ${v}_{i}$) defined as

1 $${K}_{{v}_{i}}={X}_{{v}_{i}}\prod _{{v}_{j}\in {N}_{i}}{Z}_{{v}_{j}},$$

where *X*, *Y*, *Z* are the Pauli operators. A pure graph state $|{G}_{{\boldsymbol{u}}}\rangle$, ${\boldsymbol{u}}=({u}_{1},\cdots ,{u}_{n})\in {\mathrm{\{0,}\mathrm{1\}}}^{n}$, is a common eigenstate of all stabilizer operators with

2 $${K}_{{v}_{i}}|{G}_{{\boldsymbol{u}}}\rangle =(-{\mathrm{1)}}^{{u}_{i}}|{G}_{{\boldsymbol{u}}}\rangle \,\forall {v}_{i}\in V,{u}_{i}\in \mathrm{\{0,}\,\mathrm{1\}}$$

here ${u}_{i}$ is the *i*th component of ***u*** corresponding to ${v}_{i}$, $\{|{G}_{{\boldsymbol{u}}}\rangle {\}}_{{\boldsymbol{u}}}$ forms a basis we call graph state basis. Graph state $|G\rangle$ is uniquely stabilized by ${\mathcal{K}}=\langle {\{{K}_{{v}_{i}}\}}_{{v}_{i}\in V(G)}\rangle$, namely, ${\mathcal{K}}$ is the stabilizer of $|G\rangle$, $\{{K}_{{v}_{i}}\}$ is the set of generators (that is, each element in ${\mathcal{K}}$ can be represented in this set). We say ${v}_{i}$ is the *correlation center* on which we perform *X* operation and its neighbor nodes perform *Z* operation.

An alternative description^45^ is by means of the interaction picture, where $|{G}_{{\boldsymbol{u}}}\rangle$ is created by preparing all qubits in the state $|{+}_{{\boldsymbol{u}}}\rangle =|{+}_{{u}_{1}}\rangle \otimes \cdots \otimes |{+}_{{u}_{n}}\rangle$ where $$|{+}_{u}\rangle =\{\begin{array}{ll}|+\rangle =\frac{\mathrm{|0}\rangle +\mathrm{|1}\rangle }{\sqrt{2}}, & \,{u}=\mathrm{0;}\,\\ |-\rangle =\frac{\mathrm{|0}\rangle -\mathrm{|1}\rangle }{\sqrt{2}}, & \,{u}=1.\,\end{array}$$ And then applying a control operator $C{Z}_{({v}_{i},{v}_{j})}={\prod }_{a\in {Z}_{2}}|a\rangle \langle a{|}_{{v}_{i}}\otimes {Z}_{{v}_{j}}^{a}$ for each edge $({v}_{i},{v}_{j})$, where ${v}_{i}$ is the control qubit and ${v}_{j}$ is the target qubit, that is

3 $$|{G}_{{\boldsymbol{u}}}\rangle =\prod _{({v}_{i},{v}_{j})\in E(G)}C{Z}_{({v}_{i},{v}_{j})}|{+}_{{\boldsymbol{u}}}\rangle ,$$

Through this paper, we will use both these two descriptions alternatively.

In our following schemes, keeping track of the evolution of graph state is crucial to calculate the final fidelity. Here, we will give this evolution of pure graph state under pauli operators by adjacency matrix ${\rm{\Lambda }}$. For any graph state $|{G}_{{\boldsymbol{u}}}\rangle =|{G}_{{u}_{i}{{\boldsymbol{u}}}_{{N}_{i}}{{\boldsymbol{u}}}_{{O}_{i}}}\rangle$, where ${{\boldsymbol{u}}}_{{{N}}_{{i}}}$ and ${{\boldsymbol{u}}}_{{{o}}_{{i}}}$ denote the components of ${\boldsymbol{u}}$ corresponding to nodes in ${N}_{i}$ and ${O}_{i}$, where *i* is a natural number, respectively. One can verify the following equations. For the case of single qubit pauli operations on the *i*th node ${v}_{i}$, it satisfies^46^ that

4 $${Z}_{i}|{G}_{{u}_{i}{{\boldsymbol{u}}}_{N({v}_{i})}{{\boldsymbol{u}}}_{O({v}_{i})}}\rangle =|{G}_{{\bar{u}}_{i}{{\boldsymbol{u}}}_{N({v}_{i})}{{\boldsymbol{u}}}_{O({v}_{i})}}\rangle ,$$

5 $${X}_{i}|{G}_{{u}_{i}{{\boldsymbol{u}}}_{N({v}_{i})}{{\boldsymbol{u}}}_{O({v}_{i})}}\rangle =(-{\mathrm{1)}}^{{u}_{i}}|{G}_{{u}_{i}{\bar{{\boldsymbol{u}}}}_{N({v}_{i})}{{\boldsymbol{u}}}_{O({v}_{i})}}\rangle ,$$

6 $${Y}_{i}|{G}_{{u}_{i}{{\boldsymbol{u}}}_{N({v}_{i})}{{\boldsymbol{u}}}_{O({v}_{i})}}\rangle =\iota {(-\mathrm{1)}}^{{\bar{u}}_{i}}|{G}_{{\bar{u}}_{i}{\bar{{\boldsymbol{u}}}}_{N({v}_{i})}{{\boldsymbol{u}}}_{O({v}_{i})}}\rangle .$$

where ${\bar{u}}_{i}$ denotes the bitwise complement with $\bar{0}=1$, $\bar{1}=0$, ${\bar{{\boldsymbol{u}}}}_{N({v}_{i})}=\{{\bar{u}}_{{i}_{1}},\cdots ,{\bar{u}}_{{i}_{q}}\}$ and $\iota$ is the imaginary unit. By extending above properties to the case of multiple particles, we obtain that

7 $${Z}_{\mathrm{1,}\cdots ,n}^{{\boldsymbol{e}}}|{G}_{{\boldsymbol{u}}}\rangle =|{G}_{{\boldsymbol{u}}+{\boldsymbol{e}}}\rangle ,$$

8 $${X}_{\mathrm{1,}\cdots ,n}^{{\boldsymbol{e}}}|{G}_{{\boldsymbol{u}}}\rangle =(-1{)}^{{\boldsymbol{u}}\cdot {{\boldsymbol{e}}}^{T}}|{G}_{{\boldsymbol{u}}+{\boldsymbol{e}}\cdot {\rm{\Lambda }}}\rangle ,$$

9 $${Y}_{\mathrm{1,}\cdots ,n}^{{\boldsymbol{e}}}|{G}_{{\boldsymbol{u}}}\rangle =\iota {(-1)}^{({\boldsymbol{u}}+{\boldsymbol{e}})\cdot {{\boldsymbol{e}}}^{T}}|{G}_{{\boldsymbol{u}}+{\boldsymbol{e}}(I+{\rm{\Lambda }})}\rangle .$$

where ${\boldsymbol{e}}=({e}_{1},\cdots ,{e}_{n})$, ${\boldsymbol{f}}=({f}_{1},\cdots ,{f}_{n})\in {\{\mathrm{0,}\mathrm{1\}}}^{n}$. Here ${Z}_{\mathrm{1,}\cdots ,n}^{{\boldsymbol{e}}}={{\rm{\Pi }}}_{i\mathrm{=1}}^{n}{Z}_{i}^{{e}_{i}}$ and likewise for ${X}_{\mathrm{1,}\cdots ,n}^{{\boldsymbol{e}}}$, ${Y}_{\mathrm{1,}\cdots ,n}^{{\boldsymbol{e}}}$. Eq. (7) follows immediately from Eqs (4) and (8) can be verified by direct calculation with ${\rm{\Lambda }}=({\lambda }_{ij})$. Finally, Eqs (7) and (8) imply that

10 $${({Z}^{{\boldsymbol{f}}}{X}^{{\boldsymbol{e}}})}_{\mathrm{1,}\cdots ,n}|{G}_{{\boldsymbol{u}}}\rangle =i{(-1)}^{({\boldsymbol{u}}+{\boldsymbol{f}})\cdot {{\boldsymbol{e}}}^{T}}|{G}_{{\boldsymbol{u}}+{\boldsymbol{f}}+{\boldsymbol{e}}\cdot {\rm{\Lambda }}}\rangle ,$$

thus together with $Y=\iota XZ$, Eq. (9) holds.

### MB-QNC over noiseless quantum network

#### The general MB-QNC procedures

Here, the general process of MB-QNC is systematically specified. In contrast to the standard GB-QNC, no entanglement is created during the computation, we thus have a clear distinction between the entanglement creation and its use as a resource.

The MB-QNC has several important components $(G,\,In(G),\,Out(G),\,{{\mathcal{P}}}^{M})$,

- The network graph *G*: the preparation of resource graph state $|G\rangle$. The solvability are closely related to it;
- The set of input nodes $In(G)$ and output nodes $Out(G)$: The holders and receivers of quantum information. By specifying them, the *k*-pair network problem is reduced to some quantum operations;
- Measurement pattern ${{\mathcal{P}}}^{M}$: the MB-QNC solution, it includes measurement basis and measurement order.

The main task of MB-QNC is to design a suitable measurement pattern for a given network problem. The general steps of the MB-QNC scheme are as follows:

1. Encode information into initial graph state pre-shared among all distant nodes;
2. Apply local single qubit measurements on intermediate nodes and source nodes;
3. Send the measurement results to output nodes and apply local Pauli operations to correct phases.

A specific ${G}_{k}$ network for *k* = 2 and *k* = 4 have been examined in measurement-based way^39^. And for general *k*-pair network, exact conditions should be developed in measurement-based framework. In following, we will give the sufficient conditions.

#### The sufficient conditions for solvability of general *k*-pair networks

For quantum *k*-pair problem, all source-sink pairs wish to communicate simultaneously in network with common channel. This task would equivalent to a specific unitary operation–*k* qubits permutation ${U}_{\tau }$, where $\tau$ is a bijective with $\tau :i\mapsto \tau (i)$, $\forall i\in \mathrm{\{1,}\,\cdots ,\,k\}$. $|{\psi }_{\mathrm{1,}\cdots ,k}\rangle$ is any *k* qubits state held by source nodes and output by source nodes, we have

$${U}_{\tau }|{\psi }_{\mathrm{1,}\cdots ,k}\rangle =|{\psi }_{\tau \mathrm{(1),}\cdots ,\tau (k)}\rangle .$$

Raussendorf *et al*.^43^ shows the conditions for simulating any unitary operations successfully in measurement-based way. We generalize this conclusion to quantum *k*-pair problem.

Since the permutation operation mapped pauli operators onto pauli operators under conjugation, that is,

11 $${U}_{\tau }\,{X}_{i}\,{U}_{\tau }^{\dagger }={X}_{\tau (i)},\,{U}_{\tau }\,{Z}_{i}{U}_{\tau }^{\dagger }={Z}_{\tau (i)}.$$

Therefore ${U}_{\tau }$ is Clifford group operations defined as the operations map Pauli group operators to itself under conjugation. Browne *et al*.^45^ shows that all Clifford group operations can be implemented by measurement pattern with Pauli measurements alone which are parallelized in chronological order. This makes the solvability can be examined in relatively simple measurement pattern–Pauli measurement. Let

12 $${P}_{{X}^{a}{Z}^{b}}^{{m}_{i}}=\frac{I+{(-\mathrm{1)}}^{{m}_{i}}{\iota }^{a\cdot b}{X}^{a}{Z}^{b}}{2}$$

denotes the pauli measurement on vertex ${v}_{i}$, where $a,b\in \mathrm{\{0,}\,\mathrm{1\}}$. Specifically, ${P}_{{X}^{a}{Z}^{b}}^{{m}_{i}}$ denotes $X$-basis measurement if $(a,\,b)=\mathrm{(1,}\,\mathrm{0)}$; $Y$-basis measurement if $(a,b)=\mathrm{(1,}\,\mathrm{1)}$; *Z*-basis measurement if $(a,\,b)=\mathrm{(0,}\,\mathrm{1)}$, and ${m}_{i}=\mathrm{\{0,}\,\mathrm{1\}}$ labels the measurement outcome $\{+\mathrm{1,}-\mathrm{1\}}$, respectively. Consequently, a deduced result would be obtained.

**Theorem 1**. Let ${G}_{RK}$ be a $k$-pair network graph with

13 $$G=In({G}_{RK})\cup M({G}_{RK})\cup Out({G}_{RK}),$$

14 $$In({G}_{RK})\cap M({G}_{RK})=In({G}_{RK})\cap Out({G}_{RK})=M({G}_{RK})\cap Out({G}_{RK})=\varnothing ,$$

and $|{G}_{RK}\rangle$ is a corresponding graph state. Suppose there exists a pauli measurement pattern ${{\mathcal{P}}}^{M}$ on intermediate nodes $M({G}_{RK})$ such that $|\hat{{G}_{RK}}\rangle ={{\mathcal{P}}}^{M}|{G}_{RK}\rangle$ obeys the $2k$ eigenvalue equations

15 $${X}_{{v}_{i}}{X}_{out({v}_{i})}|\hat{{G}_{RK}}\rangle =(-{\mathrm{1)}}^{{\lambda }_{x,i}}|\hat{{G}_{RK}}\rangle ,$$

16 $${Z}_{{v}_{i}}{Z}_{out({v}_{i})}|\hat{{G}_{RK}}\rangle =(-{\mathrm{1)}}^{{\lambda }_{z,i}}|\hat{{G}_{RK}}\rangle ,$$

where ${v}_{i}\in In({G}_{RK})$, ${\lambda }_{z,i},{\lambda }_{x,i}\in \mathrm{\{0,}\,\mathrm{1\}}$ are the function of measurement outcomes. Then $k$-pair network is solvable up to some local Pauli operators with the measurement pattern on $M({G}_{RK})$ and $In({G}_{RK})$ described by ${{\mathcal{P}}}^{M}$ and *X*-basis measurements respectively,

With the correlation between quantum k-pair networks and a highly entangled graph states, by reducing *k*-pair task to a specific clifford group operation, the sufficient conditions is constructed with 2*k* eigenvalue Equations (15) and (16), building an unambiguous functional relationship between the solvability and the network structure.

However, how should we design proper measurement pattern meeting these eigenvalue equations and what conditions the measurement pattern should exactly satisfies? From this point, another sufficient condition is given by the stabilizer matrix and adjacency matrix.

Note that by selecting a range of correlation centers denoted by ${{\boldsymbol{a}}}_{i}=({a}_{i1},\cdots ,{a}_{i\mathrm{,2}k+n})\in {\mathrm{\{0,}\mathrm{1\}}}^{2k+n}$, we can construct $2k$ stabilizer equations. Thus the product of these stabilizer generators are represented by

17 $${{\boldsymbol{K}}}_{{{\boldsymbol{a}}}_{i}}=\prod _{j\mathrm{=1}}^{2k+n}{({K}_{{v}_{j}})}^{{a}_{ij}},$$

with ${{\boldsymbol{K}}}_{{{\boldsymbol{a}}}_{i}}|{G}_{RK}\rangle =|{G}_{RK}\rangle$, where $i\in \{1,\cdots ,\,2k\}$. For all these ${{\boldsymbol{a}}}_{i}$, we call $$A=({a}_{ij})={(\begin{array}{c}{{\boldsymbol{a}}}_{1}\\ \vdots \\ {{\boldsymbol{a}}}_{2k}\end{array})}_{2k\times \mathrm{(2}k+n)}$$ the *stabilizer matrix*. Now, we consider the solvability with this stabilizer matrix and adjacency matrix.

**Corollary 2** Let graph ${G}_{RK}$ be a $k$-pair network with adjacency matrix ${\rm{\Lambda }}$. If stabilizer matrix $A$ satisfies the following conditions

(1)  $A$ and $B=A\cdot {\rm{\Lambda }}$ are the form of $$(\begin{array}{lll}I & \cdots  & I\\ 0 & \cdots  & 0\end{array})$$ and $$(\begin{array}{lll}{\bf{0}} & \cdots  & {\bf{0}}\\ {I} & \cdots  & {I}\end{array})$$, respectively, ***I*** and ${\bf{0}}$ are $k\times k$ identity matrix and zero matrix,

(2)  For any given column $j\in \{k+1,\cdots ,\,k+n\}$ in $A$ and $B$, $({a}_{ij},{b}_{ij})\ne 0$ implies $({a}_{tj},{b}_{tj})=({a}_{ij},{b}_{ij})$ or $({a}_{tj},{b}_{tj})=0$, $\forall t\ne i$.

Then network ${G}_{RK}$ is solvable with MB-QNC. Further, the exact parameters $a$ and $b$ in each measurement basis as in Eq. (12) can be obtained from $A$ and $B$, respectively.

Note that Theorem 1 does not imply anything about how to construct measurement basis, but Corollary 2 does by the conditions stabilizer matrix $A$ meet. It is helpful to propose specific network coding in measurement-based way. Note that Theorem 1 and Corollary 2 can also be used as the analysis of feasibility and the design of schemes for sharing EPR pairs $\frac{\mathrm{(|00}\rangle +\mathrm{|11}\rangle {)}_{{v}_{i},out({v}_{i})}}{\sqrt{2}}$ simultaneously for some communication tasks only if we do not measure input qubits since it is the unique quantum state (up to a global phase) which is stabilized by these operators ${X}_{{v}_{i}}{X}_{out({v}_{i})}$ and ${Z}_{{v}_{i}}{Z}_{out({v}_{i})}$.

#### An instance network *G*_*k*_

**Figure 3:**
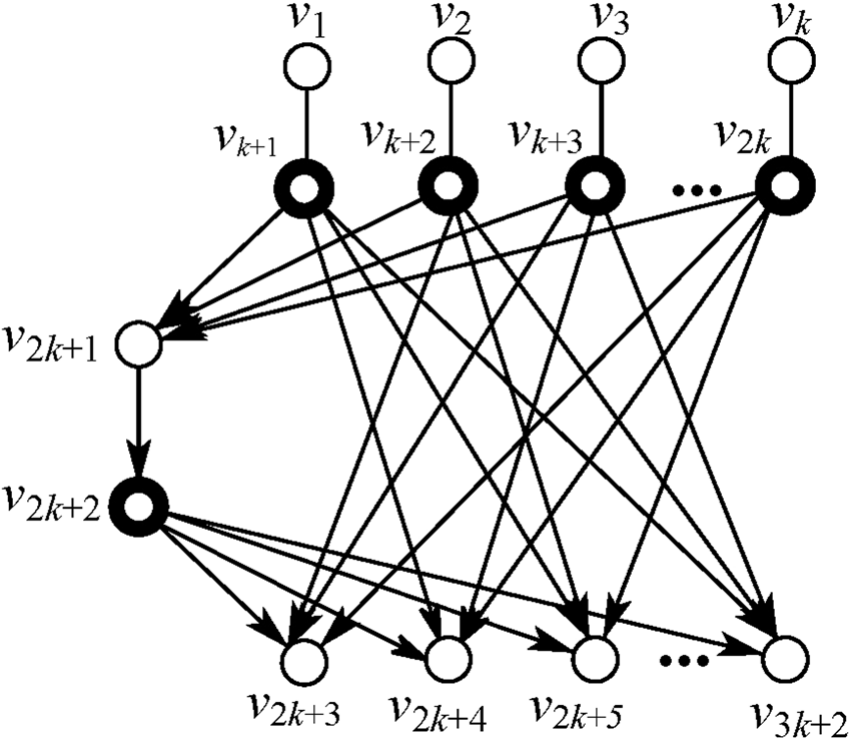
Network ${G}_{k}$ with $k$ introduced input nodes.

In the following, we apply the results developed above to an instance $k$-pair network ${G}_{k}$ as shown in Fig. 2, in which *k* source-sink pairs sharing a common channel wish to communicate with each other simultaneously. Here we introduce $k$ input nodes for quantum states to be transferred. Now, all $3k+2$ nodes are denoted by $V=\{{v}_{1},\cdots ,\,{v}_{k},{v}_{k+1},\cdots ,\,{v}_{2k},\,{v}_{2k+1},\,{v}_{2k+2},\,{v}_{2k+3},\cdots ,\,{v}_{3k+2}\}$ as shown in Fig. 3. In maths, all nodes in network graph ${G}_{k}$ can be divided into two disjoint parts ${V}_{C}=\{{v}_{k+1},\cdots ,{v}_{2k},{v}_{2k+2}\}$, ${V}_{T}=\{{v}_{1},\cdots ,{v}_{k},{v}_{2k+1},{v}_{2k+3},\cdots ,{v}_{3k+2}\}$. The solvability of ${G}_{k}$ network can be easily verified by Corollary 2.

The adjacency matrix $\Lambda$ of graph ${G}_{k}$ obtained from its partition is

18 $${\rm{\Lambda }}=\begin{array}{c}{v}_{k+1}\\ \vdots \\ {v}_{2k}\\ {v}_{2k+2}\\ {v}_{1}\\ \vdots \\ {v}_{k}\\ {v}_{2k+1}\\ {v}_{2k+3}\\ \vdots \\ {v}_{3k+2}\end{array}(\begin{array}{ccccccccccc}0 &  &  & 0 & 1 &  &  & 1 & 0 & 1\cdots  & 1\\  & \ddots  &  & \vdots  &  & \ddots  &  & \vdots  &  & \ddots  & \\  &  & 0 & 0 &  &  & 1 & 1 & 1 & \cdots 1 & 0\\ 0 & \cdots  & 0 & 0 & 0 & \cdots  & 0 & 1 & 1 & \cdots  & 1\\ 1 &  &  & 0 & 0 &  &  & 0 & 0 &  & \\  & \ddots  &  & \vdots  &  & \ddots  &  & \vdots  &  & \ddots  & \\  &  & 1 & 0 &  &  & 0 & 0 &  &  & 0\\ 1 & \cdots  & 1 & 1 & 0 & \cdots  & 0 & 0 & 0 & \cdots  & 0\\ 0 & 1\cdots  & 1 & 1 & 0 &  &  & \cdots  & 0 &  & \\  & \ddots  &  & \vdots  &  & \ddots  &  & \vdots  &  & \ddots  & \\ 1 & \cdots 1 & 0 & 1 &  &  & 0 & 0 &  &  & 0\end{array}).$$

We find a matrix $A$

19 $$\begin{array}{c}A=(\begin{array}{ccccccccccc}0 &  &  & 0 & 1 &  &  & 1 & 1 &  & \\  & \ddots  &  & \vdots  &  & \ddots  &  & \vdots  &  & \ddots  & \\  &  & 0 & 0 &  &  & 1 & 1 &  &  & 1\\ 1 &  &  & 1 & 0 &  &  & 0 & 0 &  & \\  & \ddots  &  & \vdots  &  & \ddots  &  & \vdots  &  & \ddots  & \\  &  & 1 & 1 &  &  & 0 & 0 &  &  & 0\end{array}),\\ B=A{\rm{\Lambda }}=(\begin{array}{ccccccccccc}0 &  &  & 0 & 0 &  &  & 0 & 0 &  & \\  & \ddots  &  & \vdots  &  & \ddots  &  & \vdots  &  & \ddots  & \\  &  & 0 & 0 &  &  & 0 & 0 &  &  & 0\\ 0 &  &  & 0 & 1 &  &  & 0 & 1 &  & \\  & \ddots  &  & \vdots  &  & \ddots  &  & \vdots  &  & \ddots  & \\  &  & 0 & 0 &  &  & 1 & 0 &  &  & 1\end{array}).\end{array}$$

satisfies the conditions (1) and (2). Thus ${G}_{k}$ is solvable in MB-QNC.

In fact, by matrix $A$, we can construct stabilizer equations

20 $$\begin{array}{c}{K}^{({v}_{1},{v}_{2k+1},{v}_{2k+3})}|{G}_{k}\rangle ={X}_{{v}_{1},{v}_{2k+1},{v}_{2k+3}}|{G}_{k}\rangle ,\\ \cdots \\ {K}^{({v}_{k},{v}_{2k+1},{v}_{3k+2})}|{G}_{k}\rangle ={X}_{{v}_{k},{v}_{2k+1},{v}_{3k+2}}|{G}_{k}\rangle ,\end{array}$$

21 $$\begin{array}{c}{K}^{({v}_{k+1},{v}_{2k+2})}|{G}_{k}\rangle ={X}_{{v}_{k+1},{v}_{2k+2}}{Z}_{{v}_{1},{v}_{2k+3}}|{G}_{k}\rangle ,\\ \cdots \\ {K}^{({v}_{2k},{v}_{2k+2})}|{G}_{k}\rangle ={X}_{{v}_{2k},{v}_{2k+2}}{Z}_{{v}_{k},{v}_{3k+2}}|{G}_{k}\rangle .\end{array}$$

Measure the intermediate nodes in $X$-basis, we get classical results ${s}_{k+1},\cdots ,{s}_{2k},{s}_{2k+1},{s}_{2k+2}$. The above equations induce the following eigenvalue equations for projected state $|{\hat{G}}_{k}\rangle$ by measurements on ${v}_{k+1},\cdots ,{v}_{k+n}$ with ${{\mathcal{P}}}^{M}=\{{P}_{{X}_{j}}{\}}_{j=k+\mathrm{1,}\cdots ,k+n}$, where ${P}_{{X}_{j}}=[\frac{I+{(-\mathrm{1)}}^{{s}_{j}}{X}_{j}}{2}]$, we have

22 $$\begin{array}{c}{X}_{{v}_{1},{v}_{2k+3}}|{\hat{G}}_{k}\rangle ={(-1)}^{{s}_{2k+1}}|{\hat{G}}_{k}\rangle ,\\ \cdots \\ {X}_{{v}_{k},{v}_{3k+2}}|{\hat{G}}_{k}\rangle ={(-1)}^{{s}_{2k+1}}|{\hat{G}}_{k}\rangle ,\end{array}$$

23 $$\begin{array}{c}{Z}_{{v}_{1},{v}_{2k+3}}|{\hat{G}}_{k}\rangle =(-1{)}^{{s}_{k+1}+{s}_{2k+2}}|{\hat{G}}_{k}\rangle ,\\ \cdots \\ {Z}_{{v}_{k},{v}_{3k+2}}|{\hat{G}}_{k}\rangle =(-1{)}^{{s}_{2k}+{s}_{2k+2}}|{\hat{G}}_{k}\rangle .\end{array}$$

Also with these measurement pattern, we can share $k$ EPR pairs among the $k$ source-sinks pairs simultaneously just leaving the input qubits unmeasured.

### The performance benefits of MB-QNC over noisy *k*-pair network

The transmission of quantum resource states also measurement on them will reduce the performance of long-range quantum information processing schemes. Moreover, encoding on intermediate nodes even makes things worse. Satoh^44^ shows that Gb-QNC is more sensitive to noise errors. The performance optimization in presence of noise is another concern of QNC. In this section, we explore the benefits of MB-QNC by *k*-pair communication instance ${G}_{k}$ and the merit of it is shown in terms of the output fidelity.

#### Noise model and assumptions

We mainly consider two kinds of noise sources: noisy resource states and noisy measurement caused by the errors on channel and imperfect projective measurements. Local Pauli-diagonal-noise channels are applied to described these errors. Specially, the effect on the a*th* qubit according to a probability is

24 $${\varepsilon }^{(a)}({\boldsymbol{p}})\rho =\sum _{e,f\in \mathrm{\{0,1\}}}{p}_{e,f}{X}^{e}{Z}^{f}\rho {Z}^{f}{X}^{e},$$

where $\rho$ is the density operator, ${\boldsymbol{p}}=({p}_{e,f}{)}_{e,f\in \mathrm{\{0,1\}}}=({p}_{00},{p}_{01},{p}_{10},{p}_{11})$ is the error parameter with $0\le {p}_{e,f}\le 1$, $\sum {p}_{e,f}=1$. We assume that it is independent and identically distributed on each quantum systems, *i.e*. ${\prod }_{i\mathrm{=1}}^{n}{\varepsilon }^{({v}_{i})}({\boldsymbol{p}})|G\rangle \langle G|$. That is well justified since in large-scale network the separation among the participants is large enough so that collective effects need not be taken into account. Some special channels of this kind include the bit-flip channel (with random $X$ noise) ${\varepsilon }_{X}^{(a)}({\boldsymbol{p}})$ with ${\boldsymbol{p}}=({p}_{00},\,\mathrm{0,}\,{p}_{10},\,\mathrm{0)}$, the phase-flip channel (with random $Z$ noise) ${\varepsilon }_{Z}^{(a)}({\boldsymbol{p}})$ with ${\boldsymbol{p}}=({p}_{00},{p}_{01}\mathrm{,0,0)}$, the bit-phase-flip channel here we denote as ${\varepsilon }_{X-Z}^{(a)}({\boldsymbol{p}})$ with ${\boldsymbol{p}}=({p}_{00},\,\mathrm{0,}\,\mathrm{0,}\,{p}_{11})$ and the depolarizing white-noise channel ${\varepsilon }_{D}^{(a)}({\boldsymbol{p}})$ with ${\boldsymbol{p}}=({p}_{00},{p}_{01},{p}_{10},{p}_{11})$, ${p}_{01}={p}_{10}={p}_{11}=\frac{1-{p}_{00}}{3}$. In addition, it assumes that a pre-prepared graph state according to network ${G}_{k}$ is distributed through noise channel.

#### Channel transmission errors

Generally speaking, the output fidelity of a general $k$-pair network is hard to calculate. However, we will work in stabilizer basis representation of mixed graph states by which the evolution of graph states can be easily tracked and just keeps the graph state in diagonal form. Further, we present a set of constraint conditions to obtain some simpler expressions of fidelity.

**The noisy evolution of graph state in stabilizer basis**: Let the prepared graph state be $|{G}_{{\bf{0}}}\rangle$. Since pauli operators map graph state basis to itself by properties (4)–(6), we consider mixed graph states diagonal in the graph state basis, $\rho ={\sum }_{{\boldsymbol{\mu }}}{\zeta }_{{\boldsymbol{\mu }}}|{G}_{{\boldsymbol{\mu }}}\rangle \langle {G}_{{\boldsymbol{\mu }}}|$, with ${\boldsymbol{\mu }}\in {\mathrm{\{0,}\mathrm{1\}}}^{3k+2}$, $0\,\le \,{\zeta }_{{\boldsymbol{\mu }}}\,\le \,1$ and ${\sum }_{{\boldsymbol{\mu }}}{\zeta }_{{\boldsymbol{\mu }}}=1$. We have the graph state in diagonal form^47^.

25 $$\rho =\frac{1}{{2}^{3k+2}}\sum _{{\boldsymbol{x}}}\langle {{\boldsymbol{K}}}_{{\boldsymbol{x}}}\rangle {{\boldsymbol{K}}}_{{\boldsymbol{x}}},$$

where the coefficient $\langle {{\boldsymbol{K}}}_{{\boldsymbol{x}}}\rangle ={\sum }_{\mu }{(-\mathrm{1)}}^{{\boldsymbol{\mu }}\cdot {\boldsymbol{x}}}{\zeta }_{{\boldsymbol{\mu }}}$. In this form, the fidelity can be described as

26 $$F=\langle {G}_{{\bf{0}}}|{\rho }_{G}|{G}_{{\bf{0}}}\rangle =\frac{1}{{2}^{3k+2}}\sum _{{\boldsymbol{x}}}\langle {{\boldsymbol{K}}}_{{\boldsymbol{x}}}\rangle =\sum _{{\mu },{\boldsymbol{x}}}{(-\mathrm{1)}}^{{\mu }\cdot {\boldsymbol{x}}}{\zeta }_{{\mu }}.$$

Thus the final fidelity would be closely related to the change of parameter ${\boldsymbol{x}}$. Firstly, we will describe it according the noise evolution of graph state. Since local Pauli-diagonal-noise channels on each subsystem except input qubits, ${\boldsymbol{e}}=({e}_{k+1},\,\cdots ,\,{e}_{2k},\,{e}_{2k+2},\,{0}_{1},\,\cdots ,\,{0}_{k},\,{e}_{2k+1},\,{e}_{2k+3},\,\cdots ,\,{e}_{3k+2})\in {\mathrm{\{0,}\mathrm{1\}}}^{2k+2}$ (as the set of all ${\boldsymbol{e}}$ under addition module 2 is a group which is isomorphism to ${\{\mathrm{0,}1\}}^{2k+2}$). It denotes no errors on input qubits ${v}_{1},\cdots ,{v}_{k}$ are considered, similar to ${\boldsymbol{f}}$. Accordingly, in local Pauli-diagonal-noise channels, the state evolution maps initial graph state $\rho =|{G}_{{\bf{0}}}\rangle \langle {G}_{{\bf{0}}}|$ to mixed graph state

27 $${\varepsilon }^{\mathrm{(2}k+\mathrm{2)}}({\boldsymbol{p}})\rho =\sum _{{\boldsymbol{e}},{\boldsymbol{f}}\in {\mathrm{\{0,1\}}}^{2k+2}}{\hat{P}}_{{\boldsymbol{e}},{\boldsymbol{f}}}{X}^{{\boldsymbol{e}}}{Z}^{{\boldsymbol{f}}}\rho {Z}^{{\boldsymbol{f}}}{X}^{{\boldsymbol{e}}},$$

where ${\hat{P}}_{{\boldsymbol{e}},{\boldsymbol{f}}}={\prod }_{j\mathrm{=1}}^{3k+2}{p}_{{e}_{j},{f}_{j}}$. According to Eqs (7–9), graph state basis $\{|{G}_{{\boldsymbol{\mu }}}\rangle \}$ is closed under pauli operators and only subscripts are transformed. Thus we have

28 $${\varepsilon }^{\mathrm{(2}k+\mathrm{2)}}({\boldsymbol{p}})\rho =\sum _{e{\boldsymbol{,}}f\in {\mathrm{\{0,1\}}}^{2k+2}}{\hat{P}}_{{\boldsymbol{e}},{\boldsymbol{f}}}|{G}_{{\boldsymbol{e}}\cdot {\rm{\Lambda }}+{\boldsymbol{f}}}\rangle \langle {G}_{{\boldsymbol{e}}\cdot {\rm{\Lambda }}+{\boldsymbol{f}}}|=\sum _{\mu \in {\mathrm{\{0,1\}}}^{3k+2}}{\zeta }_{\mu }|{G}_{{\boldsymbol{\mu }}}\rangle \langle {G}_{{\boldsymbol{\mu }}}\mathrm{|}.$$

Here, since ${\boldsymbol{e}}\cdot {\rm{\Lambda }}+{\boldsymbol{f}}$ traverses all value of ${\mathrm{\{0},\mathrm{1\}}}^{3k+2}$, it builds a surjection between $\{({\boldsymbol{e}},{\boldsymbol{f}})\}$ and ${\mathrm{\{0,}\mathrm{1\}}}^{3k+2}=$ $\{{\boldsymbol{e}}\cdot {\rm{\Lambda }}+{\boldsymbol{f}}\}$. Thus we have, for each ${\boldsymbol{x}}$,

29 $$\langle {{\boldsymbol{K}}}_{{\boldsymbol{x}}}\rangle =\sum _{e{\boldsymbol{,}}f}{(-\mathrm{1)}}^{{\boldsymbol{x}}\cdot ({\boldsymbol{e}}\cdot {\rm{\Lambda }}+{\boldsymbol{f}})}{\hat{P}}_{e{\boldsymbol{,}}f}.$$

Under channel noise, the evolution of graph state which embodied in the coefficient of each stabilizer element can be described for a special error parameter as in Eq. (29).

### Perfect measurement evolution in stabilizer basis

The action of Pauli measurements can also be easily described in this formalism as a transformation of the graph (up to some local unitaries) and also keep the graph state in stabilizer basis: it just multiplies each stabilizer basis by a coefficient. In terms of the stabilizer operators, the measurement of ${X}_{u}$ commutes with ${K}_{u}$, anticommutes with all ${K}_{v}$, $u\in N(v)$. Thus

30 $$[I+{(-\mathrm{1)}}^{m}{X}_{u}]{{\boldsymbol{K}}}_{{\boldsymbol{x}}}[I+{(-\mathrm{1)}}^{m}{X}_{u}]=[I+{(-\mathrm{1)}}^{m}{X}_{u}]{{\boldsymbol{K}}}_{{\boldsymbol{x}}}{\delta }_{\mathrm{0,}\sum _{v\in N(u)}{x}_{v}},$$

After tracing out qubit $u$, the new stabilizer is

31 $${(-\mathrm{1)}}^{m\cdot {x}_{u}}{\boldsymbol{K}}{^{\prime} }_{{\boldsymbol{x}}}{\delta }_{\mathrm{0,}\sum _{v\in N(u)}{x}_{v}},$$

where the new ${\boldsymbol{K}}{\text{'}}_{{\boldsymbol{x}}}$ corresponds to a new graph $G^{\prime}$ obtained from $G$ by removing or transforming the neighborhood of vertex $u$. In fact, as described in Cuquet *et al*.’s ref.^47^ measurement of $Z$ simply disconnects the measured qubit from the rest of the graph, while $X$ and $Y$ transform the neighborhood of the measured qubit and then disconnect it. Here, the phase can be corrected by ${({Z}_{u})}^{m}$ since ${({Z}_{u})}^{m}{{\boldsymbol{K}}}_{{\boldsymbol{x}}}{({Z}_{u})}^{m}=(-{\mathrm{1)}}^{m\cdot {x}_{u}}{{\boldsymbol{K}}}_{{\boldsymbol{x}}}$.

By the constraint conditions ${\delta }_{\mathrm{0,}{\sum }_{v\in N(u)}{x}_{v}}$, each measurement on subsystem will remove some certain ${{\boldsymbol{K}}}_{{\boldsymbol{x}}}$ and leave the remains ${\boldsymbol{K}}{\text{'}}_{{\boldsymbol{x}}}$ combining like terms. Here, $G^{\prime}$ is closely related the choice of neighbor node $v$ of $u$. Thus, by Hein *et al*.’s ref.^48^,

32 $$G^{\prime} =G{\rm{\Delta }}E(N(v),\,N(u)){\rm{\Delta }}E(N(v)\cap N(u),\,N(v)\cap N(u)){\rm{\Delta }}E(\{v\},\,N(u)-\{v\}),$$

where for $V^{\prime} ,V^{\prime\prime} \subset V$,$E(V^{\prime} ,V^{\prime\prime} )=\{(v,\,w)\in E|v\in V^{\prime} ,\,w\in V^{\prime\prime} ,\,v\ne w\}$, $\{u\}\cup \{v\}-$ $V^{\prime} \Delta V^{\prime\prime} =V^{\prime} \cup V^{\prime\prime} -V^{\prime} \cap V^{\prime\prime} \subset V$ $N(v)-\{u\}\cup \{others\}$. In addition, for $u$ and $v$, we can divide $V$ as $\{u\}\cup \{v\}\cup N(v)-\{u\}\cup \{others\}$ Consequently, for ${{\boldsymbol{K}}}_{{\boldsymbol{x}}}={{\boldsymbol{K}}}_{({x}_{u},{x}_{v},{{\boldsymbol{x}}}_{N(v)-\{u\}},{{\boldsymbol{x}}}_{other})}$, $X$-basis measurement on $u$ leads

**Theorem 2**. In network ${G}_{k}$, we denote ${{\boldsymbol{K}}}_{{\boldsymbol{x}}}$ as ${{\boldsymbol{K}}}_{({x}_{u},{x}_{v},{{\boldsymbol{x}}}_{N(v)-\{u\}},{{\boldsymbol{x}}}_{other})}$. Measurement with $X$-basis on $u$ leads to a new ${\boldsymbol{K}}{\text{'}}_{{\boldsymbol{x}}}$ with

33 $${\boldsymbol{K}}{\text{'}}_{{\boldsymbol{x}}}={{\boldsymbol{K}}}_{(\sum _{w\in N(v)}{x}_{w},{{\boldsymbol{x}}}_{N(v)-\{u\}},{{\boldsymbol{x}}}_{other})},$$

That is, subscript ${{\boldsymbol{x}}}_{v}$ is replaced by ${\sum }_{w\in N(v)}{x}_{w}$, ${x}_{u}$ is deleted and others remain unchanged.

**Figure 4:**
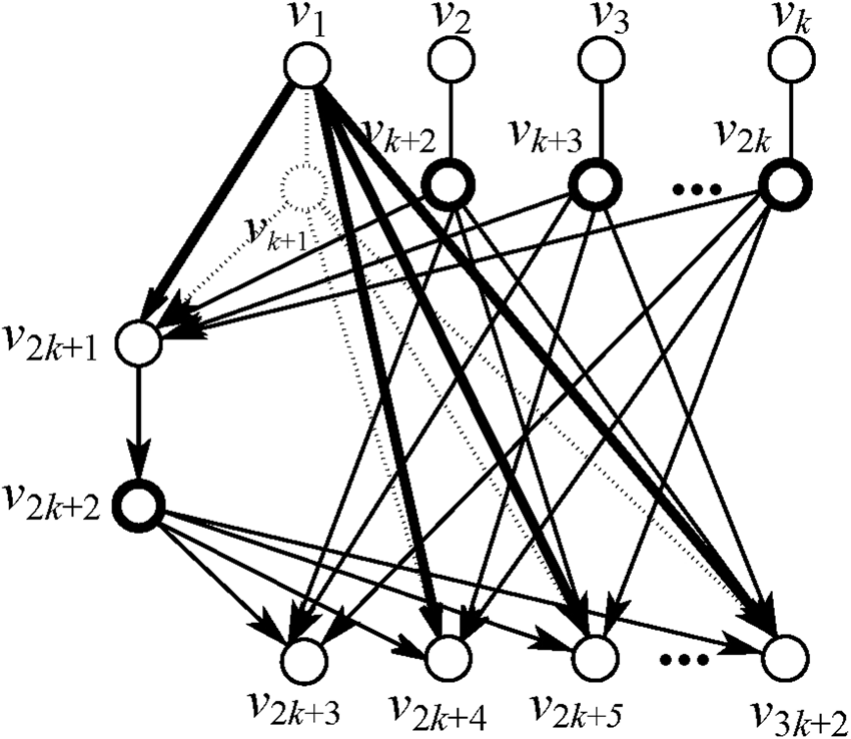
The new graph $G^{\prime}$ after measure node ${v}_{k+1}$ with $X$ **-**basis. Node ${v}_{k+1}$ and all edges connected to it is disconnected denoted by dotted lines and the transformed neighborhoods denote by bold lines.

For example, we measure node ${v}_{k+1}$ with $X$-basis, and ${v}_{1}\in N({v}_{k+1})=\{{v}_{1},{v}_{2k+1},{v}_{2k+4},\cdots ,{v}_{3k+2}\}$ as the selected neighbor node with $N({v}_{1})=\{{v}_{k+1}\}$. It implies that $E(N({v}_{k+1}),\,N({v}_{1}))\,=\,$,$\{({v}_{1},\,{v}_{k+1}),\,({v}_{2k+1},{v}_{k+1}),\,({v}_{2k+4},$ ${v}_{k+1}),\cdots ,\,({v}_{3k+2},{v}_{k+1})\}$ $N({v}_{k+1})\cap N({v}_{1})=0/$ and $E(\{{v}_{k+1}\},N({v}_{1})-\{{v}_{k+1}\})=\{({v}_{1},{v}_{2k+1}),({v}_{2k+4},{v}_{1}),\cdots ,$ $({v}_{3k+2},{v}_{1})\}$. Thus, this measurement leads a new neighborhood that build a new graph $G^{\prime}$ as shown in Fig. 4. Each ${{\boldsymbol{K}}}_{{\boldsymbol{x}}}$ is transformed as ${\boldsymbol{K}}{\text{'}}_{{\boldsymbol{x}}}={{\boldsymbol{K}}}_{({x}_{k+1},{x}_{2},\cdots ,{x}_{k},{x}_{k+2},\cdots ,{x}_{3k+2})}.$ Here, all pauli components of the measured subsystem in ${{\boldsymbol{K}}}_{{\boldsymbol{x}}}$ are deleted. Furthermore, a constraint condition can be attained, that is ${x}_{1}+{x}_{2k+1}+{x}_{2k+4}+\cdots +{x}_{3k+2}=0$. The details about the transform of ${{\boldsymbol{K}}}_{{\boldsymbol{x}}}$ and the constraint conditions can be seen in Table 1. Finally, we get the $k+2$ constraint conditions:

34 $$\begin{array}{c}{x}_{1}+{x}_{2k+1}+{x}_{2k+4}+\cdots +{x}_{3k+2}=\mathrm{0,}\\ \ddots \\ {x}_{k}+{x}_{2k+1}+{x}_{2k+4}+\cdots +{x}_{3k+1}=\mathrm{0,}\\ {x}_{2k+1}+{x}_{2k+3}+\cdots +{x}_{3k+2}=\mathrm{0,}\\ {x}_{k+1}+{x}_{2k+2}+{x}_{k+2}+\cdots +{x}_{2k}=0.\end{array}$$

**Table 1:** Transformation of subscript ${\boldsymbol{x}}$ after each measurement. After the measurement on ${v}_{k+1}$ with ${P}_{{X}_{k+1}}$, and selected neighbor node ${v}_{1}\in N({v}_{k+1})$, the initial ${{\boldsymbol{K}}}_{{x}_{1},\cdots ,{x}_{3k+2}}$ is transformed to ${{\boldsymbol{K}}}_{{x}_{k+1},{x}_{2},\cdots ,{x}_{3k+2}}$ with constraints **(** ${C}_{1}$ **)**, until the final measurement on ${v}_{2k+1}$ with ${P}_{{X}_{2k+1}}$, and selected neighbor node ${v}_{2k+3}\in N({v}_{2k+1})$, ${{\boldsymbol{K}}}_{{x}_{k+1},\cdots ,{x}_{2k},{x}_{2k+1},{x}_{2k+2}+{x}_{k+2}+\cdots +{x}_{2k},{x}_{2k+4},\cdots ,{x}_{3k+2}}$ is transformed to ${{\boldsymbol{K}}}_{{x}_{k+1},\cdots ,{x}_{2k},{x}_{2k+1}+{x}_{2k+4}+\cdots +{x}_{3k+2},{x}_{2k+4},\cdots ,{x}_{3k+2}}$ with constraints **(** ${C}_{k+2}$ **)**.

initial ${\boldsymbol{x}}$	measured nodes and basis	selected neighbor nodes	results $\bar{{\boldsymbol{x}}}$	with constraints ${{\boldsymbol{\delta }}}_{{\bf{0}},{\sum }_{{\boldsymbol{v}}\in {\boldsymbol{N}}({\boldsymbol{u}})}{{\boldsymbol{x}}}_{{\boldsymbol{v}}}}$
${x}_{1},\cdots ,{x}_{3k+2}$	${P}_{{X}_{k+1}}$	${v}_{1}$	${x}_{k+1},{x}_{2},\cdots ,{x}_{3k+2}$	$({C}_{1}){x}_{1}+{x}_{2k+1}+{x}_{2k+4}+\cdots +{x}_{3k+2}=0$
$\ddots$	$\ddots$	$\ddots$	$\ddots$	$\ddots$
${x}_{k+1},{x}_{2},\cdots ,{x}_{3k+2}$	${P}_{{X}_{2k}}$	${v}_{k}$	${x}_{k+1},\cdots ,{x}_{2k},$ ${x}_{2k+1},{x}_{2k+2},$ ${x}_{2k+3},\cdots ,{x}_{3k+2}$	$({C}_{k}){x}_{k}+{x}_{2k+1}+{x}_{2k+4}+\cdots +{{x}}_{3k+1}=0$
${x}_{k+1},\cdots ,{x}_{2k},$ ${x}_{2k+1},{x}_{2k+2},$ ${x}_{2k+3},\cdots ,{x}_{3k+2}$	${P}_{{X}_{2k+2}}$	${v}_{2k+3}$	${x}_{k+1},\cdots ,{x}_{2k},{x}_{2k+1},$ ${x}_{2k+2}+{x}_{k+2}+\cdots +{x}_{2k},$ ${x}_{2k+4},\cdots ,{x}_{3k+2}$	$({C}_{k+1}){x}_{2k+1}+{x}_{2k+3}+\cdots +{x}_{3k+2}=0$
${x}_{k+1},\cdots ,{x}_{2k},{x}_{2k+1},$ ${x}_{2k+2}+{x}_{k+2}+\cdots +{x}_{2k},$ ${x}_{2k+4},\cdots ,{x}_{3k+2}$	${P}_{{X}_{2k+1}}$	${v}_{2k+3}$	${x}_{k+1},\cdots ,{x}_{2k},$ ${x}_{2k+1}+{x}_{2k+4}+\cdots +{x}_{3k+2},$ ${x}_{2k+4},\cdots ,{x}_{3k+2}$	$({C}_{k+2}){x}_{k+1}+{x}_{2k+2}+{x}_{k+2}+\cdots +{x}_{2k}=0$

After all rounds of measurement, ${{\boldsymbol{K}}}_{{\boldsymbol{x}}}$ will be deleted as long as the conditions are not met. From the remaining, the exact fidelity associated with these constraints will be given. Denote $\bar{{\boldsymbol{x}}}$ as the subscripts satisfying these constraint conditions, the final fidelity is represented as

35 $${F}_{out}=\frac{1}{{2}^{2k}}\sum _{\bar{{\boldsymbol{x}}}}\langle {{\boldsymbol{K}}}_{\bar{{\boldsymbol{x}}}}\rangle =\frac{1}{{2}^{2k}}\sum _{\bar{{\boldsymbol{x}}},{\boldsymbol{e}},{\boldsymbol{f}}}{(-1)}^{\bar{{\boldsymbol{x}}}\cdot ({\boldsymbol{e}}\cdot {\rm{\Lambda }}+{\boldsymbol{f}})}{\hat{P}}_{e{\boldsymbol{,}}f}.$$

Specially, fidelity for bit-flip channel noise is ${F}_{out}=\frac{1}{{2}^{2k}}{\sum }_{\bar{{\boldsymbol{x}}}{\boldsymbol{,}}e}{(-\mathrm{1)}}^{\bar{{\boldsymbol{x}}}\cdot ({\boldsymbol{e}}\cdot {\rm{\Lambda }})}{\hat{P}}_{{\boldsymbol{e}},{\bf{0}}}$ with ${\boldsymbol{f}}={\bf{0}}$; for phase-flip channel noise is ${F}_{out}=\frac{1}{{2}^{2k}}{\sum }_{\bar{{\boldsymbol{x}}}{\boldsymbol{,}}f}{(-\mathrm{1)}}^{\bar{{\boldsymbol{x}}}\cdot {\boldsymbol{f}}}{\hat{P}}_{{\bf{0}},{\boldsymbol{f}}}$ with ${\boldsymbol{e}}={\bf{0}}$; for bit-phase-flip channel noise is ${F}_{out}=\frac{1}{{2}^{2k}}{\sum }_{\bar{{\boldsymbol{x}}},{\boldsymbol{e}},}{(-1)}^{\bar{{\boldsymbol{x}}}\cdot ({\boldsymbol{e}}\cdot {\rm{\Lambda }}+{\boldsymbol{e}})}{\hat{P}}_{e{\boldsymbol{,}}e}$; and for depolarizing white-noise channel noise is ${F}_{out}=\frac{1}{{2}^{2k}}{\sum }_{\bar{{\boldsymbol{x}}},{\boldsymbol{e}},{\boldsymbol{f}}}{(-1)}^{\bar{{\boldsymbol{x}}}\cdot ({\boldsymbol{e}}\cdot {\rm{\Lambda }}+{\boldsymbol{f}})}{\hat{P}}_{{\boldsymbol{e}},{\boldsymbol{f}}}$ with ${p}_{01}={p}_{10}={p}_{11}=\frac{1-{p}_{00}}{3}$.

#### Imperfectly local operations: the evolution of measurement in stabilizer basis

Another noise resource we consider is the imperfectly local measurement. Here, noisy measurements ${\hat{P}}_{{X}^{a}{Z}^{b}}^{{m}_{i}}$ can be described by a two-step process, a perfect measurement ${P}_{{X}^{a}{Z}^{b}}^{{m}_{i}}$ is applied after noise acts on all particles that are subjected to the measurement, that is, ${\tilde{P}}_{{X}^{a}{Z}^{b}}^{{m}_{i}}={P}_{{X}^{a}{Z}^{b}}^{{m}_{i}}{\varepsilon }^{({v}_{i})}$. Therefore, in the presence of imperfect operation, the Eq. (35) still holds.

For the case of both these two noises, an important property is that multiple noises on the same qubit are equal to the linear superposition of multiple error parameters^49^, that is, ${\varepsilon }^{({v}_{i})}({{\boldsymbol{p}}}_{1}){\varepsilon }^{({v}_{i})}({{\boldsymbol{p}}}_{2})={\varepsilon }^{({v}_{i})}({\boldsymbol{p}}{\rm{^{\prime} }})$, where ${\boldsymbol{p}}{\rm{^{\prime} }}$ is a linear function of the channel noise ${{\boldsymbol{p}}}_{1}$ and imperfectively operation noise ${{\boldsymbol{p}}}_{2}$. Therefore, one can summarize the effect of all noises by a single noisy channel ${\varepsilon }^{({v}_{i})}({\boldsymbol{p}}{\rm{^{\prime} }})$, acting on all the network nodes(except the input nodes) should be understood as representing all kinds of imperfections in noisy resource state and noisy measurements. The final fidelity is represented as

36 $${F}_{out}=\sum _{\bar{{\boldsymbol{x}}}}\langle {{\boldsymbol{K}}}_{\bar{{\boldsymbol{x}}}}\rangle =\sum _{\bar{{\boldsymbol{x}}},{\boldsymbol{e}},{\boldsymbol{f}}}{(-1)}^{\bar{{\boldsymbol{x}}}\cdot ({\boldsymbol{e}}\cdot {\rm{\Lambda }}+{\boldsymbol{f}})}\hat{P}{\text{'}}_{{\boldsymbol{e}},{\boldsymbol{f}}},$$

#### Performance Comparision

Compared with GB-QNC in Satoh *et al*.’s scheme^44^, the MB-QNC has significant advantage in the final fidelity which can be shown in the task of sharing EPR pairs in $k$-pair network. We assume the same error $p=1-{p}_{00}$ that each single subsystem suffers. Consider network ${G}_{k}$ instance for $k=2$. Firstly, by the Eq. (34), we get the constraint condition

37 $$\begin{array}{c}{x}_{1}+{x}_{4}+{x}_{6}=\mathrm{0,}{x}_{2}+{x}_{4}+{x}_{8}=\mathrm{0,}\\ {x}_{4}+{x}_{6}+{x}_{8}=\mathrm{0,}{x}_{3}+{x}_{5}+{x}_{7}=0.\end{array}$$

Thus we have all $\bar{{\boldsymbol{x}}}$ meet Eq. (37) in Table 2. When $Z$ errors exist with $p$, we have ${p}_{00}=1-p$ and ${p}_{01}=p$. The fidelity can be calculated by

38 $${F}_{out}=\sum _{\bar{{\boldsymbol{x}}},{\boldsymbol{f}}}{(-\mathrm{1)}}^{\bar{{\boldsymbol{x}}}\cdot {\boldsymbol{f}}}{\hat{P}}_{{\bf{0}},{\boldsymbol{f}}}\mathrm{=(1}-3p+3{p}^{2}{)}^{2}.$$

For random $X$ errors, ${p}_{00}=1-p$ and ${p}_{10}=p$. The fidelity can be calculated by

39 $${F}_{out}=\sum _{\bar{{\boldsymbol{x}}},{\boldsymbol{f}}}{(-\mathrm{1)}}^{\bar{{\boldsymbol{x}}}\cdot ({\boldsymbol{e}}\cdot {\rm{\Lambda }})}{\hat{P}}_{{\boldsymbol{e}},{\bf{0}}}=1-2p+{p}^{2}.$$

**Table 2:** All ${\boldsymbol{x}}$ meet the constraint conditions in Eq. (37).

$$\bar{{\boldsymbol{x}}}=({x}_{3},{x}_{5},{x}_{7},{x}_{1},{x}_{2},{x}_{4},{x}_{6},{x}_{8})$$			
00000000	00001110	00010101	00011011
01100000	01101110	01110101	01111011
10100000	10101110	10110101	10111011
11000000	11001110	11010101	11011011

In fact, for some ${\boldsymbol{f}}$ and ${\boldsymbol{e}}$, ${\hat{P}}_{{\bf{0}},{\boldsymbol{f}}}$, ${\hat{P}}_{{\boldsymbol{e}},{\bf{0}}}$ will be deleted as the cancellation coefficient and the remaining can be seen in Table 3. Consequently, we get Eqs (38) and (39).

**Table 3:** The parameters ***f*** and ***e*** keep ${\hat{P}}_{{\bf{0}},{\boldsymbol{f}}}$, ${\hat{P}}_{{\boldsymbol{e}},{\bf{0}}}$. In Eqs (38) and (39), constant coefficients ${\sum }_{\bar{{\boldsymbol{x}}},{\boldsymbol{f}}}{(-1)}^{\bar{{\boldsymbol{x}}}\cdot {\boldsymbol{f}}}$ equal to 0 as the cancellation coefficient leading some ${\hat{P}}_{{\bf{0}},{\boldsymbol{f}}}$, ${\hat{P}}_{{\boldsymbol{e}},{\bf{0}}}$ cancelled. Take ${\boldsymbol{f}}\mathrm{=11100000}$ and $01100000$ for example which are belong and not belong to Table 3. ${\sum }_{\bar{{\boldsymbol{x}}}}{(-1)}^{\bar{{\boldsymbol{x}}}\cdot \mathrm{(11100000)}}={2}^{4}$ and ${\sum }_{\bar{{\boldsymbol{x}}}}{(-\mathrm{1)}}^{\bar{{\boldsymbol{x}}}\cdot \mathrm{(01100000)}}=0$.

${\boldsymbol{e}}$				${\boldsymbol{f}}$
00000000	00100000	01000000	01100000	00000000
10000000	10100000	11000000	11100000	00000111
00000100	00100100	01000100	01100100	11100000
10000100	10100100	11000100	11100100	11100111

**Figure 5:**
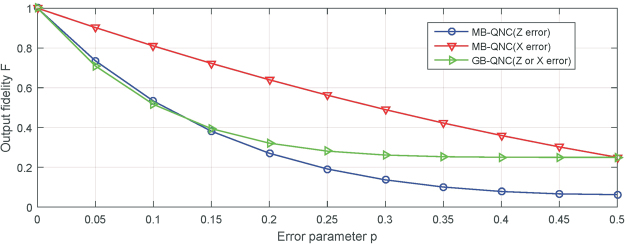
Compared with the GB-QNC in Satoh *et al*.’s scheme. For *X* error, the error threshold is about 30% in MB-QNC is significantly better than in GB-QNC 10%. And the threshold for *Z* error, it is slightly better.

By comparing with the GB-QNC in Satoh *et al*.’s scheme, as shown in Fig. 5, MB-QNC allows higher error threshold. For *X* error, the error threshold is about 30% in MB-QNC is significantly better than in GB-QNC 10%. And the threshold for *Z* error, it is slightly better than it.

## Discussion

In this paper, we firstly present sufficient conditions for the solvability of general quantum *k*-pair network with MB-QNC. It solve the central question in quantum communication whether a given network usually with capacity constraints can handle a specific joint communication task. With the correlation between quantum *k*-pair networks and a highly entangled graph states, the sufficient conditions is constructed by 2 *k* eigenvalue Equations (15) and (16). Thus a quantifiable relationship between solvability and network structure is built. Further, for the question how to construct coding scheme and what conditions the coding operations should exactly satisfies? We present another sufficient condition given by the stabilizer matrix and adjacency matrix. It would helpful in designing specific coding scheme for quantum communication tasks to attain high capacity. We find that it would be also helpful in constructively analyze the feasibility to sharing *k* EPR pairs over arbitrary networks. Finally, we show that in the same initial error parameter, MB-QNC allows higher error threshold compared with the existing GB-QNC. Thus, we optimize transmission schemes to better defend the effects of noisy resource states and noisy measurement caused by channel errors and imperfect local operations. Take an instance network ${G}_{k}$, the analysis shows that for *X* error, the error threshold about 30% in MB-QNC is significantly better than in GB-QNC 10%. And the threshold for *Z* error is slightly better. This conclusion can be extended to any quantum *k*-pair network who has a classical linear network code since it is in effect a measurement-based procedure which performs only *X*-eigenbasis measurements, on a graph state with similar structure to the coding network.

In experimental implementations, despite the fact that projective measurement involved in measurement-based way can nowadays be implemented, the hardest task from experimental point of view is the distribution of graph states in large scale quantum network. In general, the fidelity drops exponentially with the growth of network size. At present, entanglement purification^50^ and entanglement distillation^51^ are two main techniques to improve the initial fidelity of the prepared entanglement states. For arbitrary (complex) network, the schemes for graph states distribution in the presence of noise have been implemented efficiently^47^. So, our scheme would be implemented in experiment with the current quantum techniques. This also makes MB-QNC schemes very attractive from an experimental perspective.

## Method

### **Proof of Corollary 2**

In order to prove Corollary 2, we construct $2k$ stabilizer equations with row vectors ${{\boldsymbol{a}}}_{1},\cdots ,{{\boldsymbol{a}}}_{2k}$ of matrix $A$ as follow:

40 $${{\boldsymbol{K}}}_{{{\boldsymbol{a}}}_{i}}|{G}_{RK}\rangle =\prod _{j\mathrm{=1}}^{2k+n}{X}_{{v}_{j}}^{{a}_{ij}}{Z}_{{v}_{j}}^{{b}_{ij}}|{G}_{RK}\rangle ,$$

where ${b}_{ij}={{\boldsymbol{a}}}_{i}\cdot {{\rm{\Lambda }}}_{j}$ denotes the total numbers of performing pauli $Z$ operation on ${v}_{j}$ with ${{\rm{\Lambda }}}_{j}$ is the $j$ th column of ${\rm{\Lambda }}$ and. Since the former and the late $k$ columns of $A$ and $A\cdot {\rm{\Lambda }}$ have the form $$(\begin{array}{c}{I}\\ {\bf{0}}\end{array})$$ and $$(\begin{array}{c}{\bf{0}}\\ {I}\end{array})$$ by condition (1), the stabilizer equations can be rewrite as

41 $${{\boldsymbol{K}}}_{{{\boldsymbol{a}}}_{i}}|{G}_{RK}\rangle ={X}_{{v}_{i}}{X}_{out({v}_{i})}\prod _{j=k+1}^{k+n}{X}_{{v}_{j}}^{{a}_{ij}}{Z}_{{v}_{j}}^{{b}_{ij}}|{G}_{RK}\rangle ,$$

42 $${{\boldsymbol{K}}}_{{{\boldsymbol{a}}}_{k+i}}|{G}_{RK}\rangle ={Z}_{{v}_{k+i}}{Z}_{out({v}_{k+i})}\prod _{j=k+1}^{k+n}{X}_{{v}_{j}}^{{a}_{k+i,j}}{Z}_{{v}_{j}}^{{b}_{k+i,j}}|{G}_{RK}\rangle .$$

Now we construct measurement basis from $A$ and $B$. First, define $\eta :{Z}_{2}^{2k}\to {Z}_{2}$ as $$\eta (\hat{{\boldsymbol{x}}})=\{\begin{array}{ll}\mathrm{0,} & \hat{{\boldsymbol{x}}}=\mathrm{0;}\\ \mathrm{1,} & {\rm{otherwise}}.\end{array}$$ “$\hat{{\boldsymbol{x}}}$” denote the column vector of a matrix. It is obviously that for all $\hat{{\boldsymbol{a}}}$, $\eta (\hat{{\boldsymbol{a}}}\mathrm{)=1}$. We claim that ${\{{P}_{{X}^{\eta ({\hat{{\boldsymbol{a}}}}_{j})}{Z}^{\eta ({\hat{{\boldsymbol{b}}}}_{j})}}\}}_{j\in \{k+\mathrm{1,}\cdots ,k+n\}}$ is the desired measurement pattern. It can be verified by performing on all $2k$ stabilizer equations. By condition (2), $(\eta ({\hat{{\boldsymbol{a}}}}_{j}),\eta ({\hat{{\boldsymbol{b}}}}_{j}))=({a}_{ij},\,{b}_{ij})$ or $({a}_{ij},{b}_{ij})\,=\,0$, thus we have

43 $$\prod _{j=k+1}^{k+n}{P}_{{X}^{\eta ({\hat{{\boldsymbol{a}}}}_{j})}{Z}^{\eta ({\hat{{\boldsymbol{b}}}}_{j})}}\prod _{j=k+1}^{k+n}{X}_{{v}_{j}}^{{a}_{ij}}{Z}_{{v}_{j}}^{{b}_{ij}}={(-1)}^{{\lambda }_{i}}\prod _{j=k+1}^{k+n}{P}_{{X}^{\eta ({\hat{{\boldsymbol{a}}}}_{j})}{Z}^{\eta ({\hat{{\boldsymbol{b}}}}_{j})}},$$

which implies the Eqs (15 and 16). Therefore, $k$-pair network ${G}_{RK}$ is solvable by Theorem 1.

### **Proof of Theorem 2**

In order to study the transformation of the subscript after measurement, we will discuss each component of the Eq. (32). First, it is easy to verified that the component $E(N(v)\cap N(u),\,N(v)\cap N(u))=0/$ since ${G}_{k}$ is a bigraph. Thus, Eq. (32) can be reduced to

44 $$G^{\prime} =G{\rm{\Delta }}E(N(u),N(v)){\rm{\Delta }}E(\{v\},N(u)-\{v\}),$$

Also by the bigraph property, we have

45 $$E(\{v\},N(u)-\{v\})\cap G=0/,$$

And for $E(N(u),N(v))$, we have

46 $$E(N(u),N(v))=E(\{u\},N(u))\cup E(\{v\},N(v)-\{u\})\cup E(N(v)-\{u\},N(u)-\{v\}),$$

Here,

47 $$E(\{u\},N(u)),E(\{v\},N(v)-\{u\})\subseteq G.$$

$E(N(v)-\{u\},N(u)-\{v\})$ is divided into two disjoint set,

48 $$\begin{array}{rcl}E(N(v)-\{u\},N(u)-\{v\}) & = & \mathop{\cup }\limits_{c\in N(u)-\{v\}}E(\{c\},N(c)\cap (N(v)\\  &  & -\,\{u\}))\cup E(\{c\},(N(v)-\{u\})\\  &  & -\,N(c)\cap (N(v)-\{u\mathrm{\})).}\end{array}$$

As mentioned above, the measurement on $u$ transforms $G$ to $G^{\prime}$ where node $u$ and edges connected to it will be deleted. Meanwhile, with these changes, some edges will be set up. Specially,

49 $$E(\{u\},N(u)),E(\{v\},N(v)-\{u\}),\mathop{\cup }\limits_{c\in N(u)-\{v\}}E(\{c\},N(c)\cap (N(v)-\{u\})),$$

will be deleted and

50 $$E(\{v\},N(u)-\{v\}),\mathop{\cup }\limits_{c\in N(u)-\{v\}}E(\{c\},(N(v)-\{u\})-N(c)\cap (N(v)-\{u\})),$$

will be set up.

Next, we will compute the subscript changes in the ${{\boldsymbol{K}}}_{{\boldsymbol{x}}}$. Note that the change of adjacency relation is only shown between the nodes and $N(u),N(v)$, and other nodes do not change. Therefore, we only consider the transformation from Eqs (49) to (50). We have, for any ${w}_{1}\in N(u)$, ${w}_{2}\in N(v)$,

51 $$\begin{array}{c}{{\boldsymbol{K}}}_{{\boldsymbol{x}}}={{\boldsymbol{K}}}_{({x}_{u},{x}_{v},{{\boldsymbol{x}}}_{N(u)-\{v\}},{{\boldsymbol{x}}}_{N(v)-\{u\}},{{\boldsymbol{x}}}_{other})}\\ \,\,\,\,\,=({X}_{u}{)}^{{x}_{u}}{({Z}_{u})}^{\sum _{{w}_{1}\in N(u)}{x}_{{w}_{1}}}{({X}_{v})}^{{x}_{v}}{({Z}_{v})}^{\sum _{{w}_{2}\in N(v)}{x}_{{w}_{2}}}\\ \prod _{{w}_{1}\in N(u)-\{v\}}{({X}_{{w}_{1}})}^{{x}_{{w}_{1}}}{({Z}_{{w}_{1}})}^{\sum _{{w}_{2}\in N({w}_{1})\cap (N(v)-\{u\})}{x}_{{w}_{2}}}{(\prod _{{w}_{1}\in N(u)-\{v\}}{Z}_{{w}_{1}})}^{{x}_{u}}\\ \prod _{{w}_{2}\in N(v)-\{u\}}{({X}_{{w}_{2}})}^{{x}_{{w}_{2}}}{({Z}_{{w}_{2}})}^{\sum _{{w}_{1}\in N({w}_{2})\cap (N(u)-\{v\})}{x}_{{w}_{1}}}{(\prod _{{w}_{2}\in N(v)-\{u\}}{Z}_{{w}_{2}})}^{{x}_{v}},\end{array}$$

and

52 $$\begin{array}{c}{{\boldsymbol{K}}}_{{\boldsymbol{x}}}={{\boldsymbol{K}}}_{({x}_{u},{x}_{v},{{\boldsymbol{x}}}_{N(u)-\{v\}},{{\boldsymbol{x}}}_{N(v)-\{u\}},{{\boldsymbol{x}}}_{other})}\\ \,\,\,\,\,\,=({X}_{u}{)}^{{x}_{u}}{({Z}_{u})}^{\sum _{{w}_{1}\in N(u)}{x}_{{w}_{1}}}{({X}_{v})}^{{x}_{v}}{({Z}_{v})}^{\sum _{{w}_{2}\in N(v)}{x}_{{w}_{2}}}\\ \prod _{{w}_{1}\in N(u)-\{v\}}{({X}_{{w}_{1}})}^{{x}_{{w}_{1}}}{({Z}_{{w}_{1}})}^{\sum _{{w}_{2}\in N({w}_{1})\cap (N(v)-\{u\})}{x}_{{w}_{2}}}{(\prod _{{w}_{1}\in N(u)-\{v\}}{Z}_{{w}_{1}})}^{{x}_{u}}\\ \prod _{{w}_{2}\in N(v)-\{u\}}{({X}_{{w}_{2}})}^{{x}_{{w}_{2}}}{({Z}_{{w}_{2}})}^{\sum _{{w}_{1}\in N({w}_{2})\cap (N(u)-\{v\})}{x}_{{w}_{1}}}{(\prod _{{w}_{2}\in N(v)-\{u\}}{Z}_{{w}_{2}})}^{{x}_{v}},\end{array}$$

Similarly, we have

53 $$\begin{array}{c}\prod _{{w}_{2}\in N(v)-\{u\}}{({Z}_{{w}_{2}})}^{\sum _{{w}_{1}\in N({w}_{2})\cap (N(u)-\{v\})}{x}_{{w}_{1}}}{(\prod _{{w}_{2}\in N(v)-\{u\}}{Z}_{{w}_{2}})}^{{x}_{v}}\\ =\,{(\prod _{{w}_{2}\in N(v)-\{u\}}{Z}_{{w}_{2}})}^{\sum _{{w}_{1}\in N(u)}{x}_{{w}_{1}}}\prod _{{w}_{2}\in N(v)-\{u\}}{({Z}_{{w}_{2}})}^{\sum _{{w}_{1}\in (N(v)-\{u\})-N({w}_{2})\cap (N(v)-\{u\})}{x}_{{w}_{1}}}\\ =\,\prod _{{w}_{2}\in N(v)-\{u\}}{({Z}_{{w}_{2}})}^{\sum _{{w}_{1}\in (N(v)-\{u\})-N({w}_{2})\cap (N(v)-\{u\})}{x}_{{w}_{1}}}.\end{array}$$

since ${\sum }_{{w}_{1}\in N(u)}{x}_{{w}_{1}}\,=\,0$. Note that by $HZXH=XZ$, ${H}_{v}{({X}_{v})}^{{x}_{v}}{({Z}_{v})}^{{\sum }_{{w}_{2}\in N(v)}{x}_{{w}_{2}}}{H}_{v}=({Z}_{v}{)}^{{x}_{v}}{({X}_{v})}^{{\sum }_{{w}_{2}\in N(v)}{x}_{{w}_{2}}}$. Thus Eq. (51) is transformed to

$$\begin{array}{c}{{\boldsymbol{K}}}_{{\boldsymbol{x}}}=[({X}_{u}{)}^{{x}_{u}}{({Z}_{u})}^{\sum _{{w}_{1}\in N(u)}{x}_{{w}_{1}}}]({X}_{v}{)}^{\sum _{{w}_{2}\in N(v)}{x}_{{w}_{2}}}\prod _{{w}_{1}\in N(u)-\{v\}}{({X}_{{w}_{1}})}^{{x}_{{w}_{1}}}\prod _{{w}_{2}\in N(v)-\{u\}}{({X}_{{w}_{2}})}^{{x}_{{w}_{2}}}\\ \left[{({Z}_{v})}^{\sum _{{w}_{1}\in N(u)-\{v\}}{x}_{{w}_{1}}}{(\prod _{{w}_{1}\in N(u)-\{v\}}{Z}_{{w}_{1}})}^{\sum _{{w}_{2}\in N(v)}{x}_{{w}_{2}}}\right]\\ \left[{({Z}_{{w}_{1}})}^{\sum _{{w}_{2}\in N({w}_{1})\cap (N(v)-\{u\})}{x}_{{w}_{2}}}{(\prod _{{w}_{1}\in N(u)-\{v\}}{Z}_{{w}_{1}})}^{{x}_{u}}{({Z}_{{w}_{2}})}^{\sum _{{w}_{1}\in N({w}_{2})\cap (N(u)-\{v\})}{x}_{{w}_{1}}}{(\prod _{{w}_{2}\in N(v)-\{u\}}{Z}_{{w}_{2}})}^{{x}_{v}}\right].\end{array}$$

Although here Hadamard operator is introduced, it does not affect the final fidelity value, which can be seen from the expression of fidelity. Therefore, we the Eq. (33) holds.
